# Health Beliefs regarding Dietary Behavior and Physical Activity of Surinamese Immigrants of Indian Descent in The Netherlands: A Qualitative Study

**DOI:** 10.5402/2012/903868

**Published:** 2012-11-27

**Authors:** A.-M. Hendriks, J. S. Gubbels, M. W. J. Jansen, S. P. J. Kremers

**Affiliations:** ^1^Academic Collaborative Centre for Public Health Limburg, Regional Public Health Service, Geleen, The Netherlands; ^2^CAPHRI School of Public Health and Primary Care, Maastricht University, P.O. Box 616, 6200 MD Maastricht, The Netherlands; ^3^Department of Health Promotion, Faculty of Health, Medicine and Life Sciences, Maastricht University, P.O. Box 616, 6200 MD Maastricht, The Netherlands; ^4^NUTRIM School for Nutrition, Toxicology and Metabolism, Maastricht University, P.O. Box 616, 6200 MD Maastricht, The Netherlands; ^5^Department of Health Services Research, Faculty of Health, Medicine and Life Sciences, Maastricht University, 6200 MD Maastricht, The Netherlands

## Abstract

This study explored the health beliefs about eating habits and physical activity (PA) of Surinamese immigrants of Indian (Hindustani) descent to examine how health education messages to prevent obesity can be made more culturally sensitive. Indians are known for their increasing obesity incidence and are highly vulnerable for obesity-related consequences such as cardiovascular diseases and diabetes. Therefore they might benefit from culturally sensitive health education messages that stimulate healthy eating habits and increase PA levels. In order to examine how health education messages aimed at preventing obesity could be adapted to Indian culture, we interviewed eight Hindustanis living in The Netherland, and conducted two focus groups (*n* = 19) with members from a Surinamese Hindustani community. Results showed cultural implications that might affect the effectiveness of health education messages: karma has a role in explaining the onset of illness, traditional eating habits are perceived as difficult to change, and PA was generally disliked. We conclude that health education messages aimed at Hindustani immigrants should recognize the role of karma in explaining the onset of illness, include more healthy alternatives for traditional foods, pay attention to the symbolic meaning of food, and suggest more enjoyable and culturally sensitive forms of PA for women.

## 1. Introduction

Overweight and obesity is increasing worldwide more rapidly than ever, also among the Indian population [1–6]. For example, overweight and obesity among women in India increased with 24.52% between 1998 and 2006 [5]. This trend is especially prevalent among high-socioeconomic groups that live in urban areas [3]. Besides, people of Indian origin have the highest prevalence of diabetes and cardiovascular diseases (CVD) in the world, which are related to genetic predispositions and amplified by the increase in current Western lifestyles and obesity [7–19]. Moreover, prevalence of CVD and diabetes among Indian *immigrants* in other countries even exceeds that of the indigenous populations and Indians living in India [12–20]. This is unusual since the prevalence of noncommunicable diseases among immigrants generally lies between those of the country of origin and the country of adoption [14]. The mortality from CVD and prevalence of diabetes are much higher among Indian immigrants compared to other ethnic groups [12–20]. In The Netherlands too, these ethnic disparities between immigrants of Indian origin and the indigenous Dutch population are apparent [7, 12, 20]. Moreover, these disparities are currently increasing, since the majority of Indian immigrants living in The Netherlands are now in their fifties, so obesity-related vulnerabilities (such as CVD and diabetes) are increasingly manifesting themselves. These immigrants have been coming to The Netherlands from Suriname since that country started to move towards independence from the Dutch, a development which started in 1954 [21]. They now form the second largest subgroup among the Surinamese immigrants living in The Netherlands (after those of African descent). These immigrants have ancestors who emigrated to Suriname from Northern India in the nineteenth century, and since this part of India is known as Hindustan, these immigrants are also known as “Surinamese Hindustanis” or “Hindustanis” (referred to below as Hindustanis). These Hindustanis immigrated to Suriname to work on the plantations during the late 19th century and early 20th century, replacing the former African slaves. After Suriname became independent in 1975, more than 40,000 of these Hindustanis decided to move to The Netherlands due to increased ethnic tensions in Suriname. Currently, 37% of the Surinamese population still consists of Hindustanis [22, 23], and they form the second largest immigrant group from Suriname in The Netherlands, estimated to include 160,000 people [23].

To date, very little is known about ways to adapt health education messages to the needs of the Hindustanis living in The Netherlands [24], whereas investing in efforts to design culturally sensitive health education messages to prevent obesity among Hindustanis is now both more legitimate and more urgent than ever.

Prevention of obesity ideally focuses on “energy balance-related behaviors”, that is, both dietary behavior and physical activity (PA) [25]. Those behaviors might be affected by personal, cultural, environmental, and societal factors. Dietary behavior might, for example, be related to cultural specific phenomena such as the symbolic meaning of traditional eating habits for immigrants [26], and the opportunity to become physically active might be affected by gender-specific phenomena. Farooqi et al. [27], for example, found that the lack of women-only exercise facilities acted as a barrier to preventive actions in Indians living in the United Kingdom, but only in women. These cultural implications can result in small but chronic energy imbalances leading to obesity on the long-term and might affect the effectiveness of health education messages regarding dietary behavior and physical activity. For example, a recent study among Surinamese living in The Netherlands showed that beliefs regarding dietary behaviors were based on deep-rooted cultural beliefs and values, and therefore very difficult to change [26]. Other studies also found such culturally determined factors influencing health behaviors: a lack of PA has been found to be relatively common among Indians [28–30]; Indians lack information about how to cook their traditional food more healthily [27], attach an important symbolic meaning to traditional eating habits [31], and feel they are not very susceptible to develop CVD and also tend to underestimate the seriousness of CVD (which is an important obesity-related consequence) [32]. Moreover, previous studies have shown that Indians may attribute and explain illness in terms of chance-related factors, fate or karma [33–35]; they may use different coping strategies to deal with symptoms and illness [36–38], and Indian immigrants' health beliefs are little affected by acculturation (i.e., they preserve their traditions) [26, 31, 39, 40]. Furthermore, the motivations to comply with an “unhealthy” subjective norm may function as a barrier to preventive action since a strong influence of cultural norms is thought to be much more embedded in behavior in collectivistic cultures such as the Indian culture. This makes it more difficult for Indians to participate in health behaviors that differ from the social norm than for people from individualistic cultures [41–43]. To examine if these factors are also salient for Hindustanis living in The Netherlands and whether there might be other cultural factors influencing this group, we qualitatively explored the health beliefs regarding eating habits and PA of this community. This knowledge can be used to make interventions created for the general population more culturally tailored to Hindustanis living in The Netherlands.

## 2. Methodology

Since culture is transmitted through language and the everyday interaction of individuals, a combination of semistructured interviews and focus groups was regarded as the most appropriate way of assessing health beliefs relevant for eating habits and PA among Surinamese Hindustanis living in The Netherlands.

### 2.1. Theoretical Framework

To structure and analyze our interviews, we used a theoretical framework consisting of the health belief model (HBM) and theory of planned behavior (TPB). The HBM and the TPB have been argued to be the most relevant social cognitive models to assess health beliefs [44–46]. The interview protocol was therefore mainly based on these two models.

The HBM [45] has three main components: perceived threat, outcome expectations and cues to action. Raising awareness, and increasing the perceived threat of obesity or related consequences such as diabetes, combined with improving knowledge, reducing barriers, and therefore improving outcome expectations, are expected to increase the adoption of healthy lifestyles. Cues to action, such as family members suffering from obesity-related consequences, are also expected to improve health behaviors. Together, these cultural affected components increase the likelihood of preventive actions being taken, especially when self-efficacy for such behaviors is high [47].

The TPB [46] may explain the effects of certain attitudes, subjective norms, and perceived behavioral control and consequently the intention to make lifestyle changes. Attitudes, subjective norms (including motivation to comply), and perceived behavioral control among Indian people with regard to lifestyle are expected to be different from those among Western people. For example, physical activity might be evaluated as a necessary activity rather than a leisure time activity in cultures that have evolved in countries where the climate is very humid or hot [48].

### 2.2. Study Participants

The study population consisted of Hindustani community members living in The Hague, The Netherlands. In total, 27 Hindustanis were interviewed (Table 1): eight during individual interviews and 19 during two focus groups (consisting of resp., ten and nine respondents). All respondents were asked to report their health status (Table 2) (these data were thus dependent on the participants' willingness to share this information). The participants had been living in The Netherlands for 25 to 39 years and self-identified themselves as being of Hindustani origin. They also felt 100% Hindu. Their Hindustani identity was expressed through or reflected in their lifestyle behaviors, such as cooking traditional Hindustani food, their religious practices, and the courses on Hinduism they oblige their own children or grandchildren to attend. They emphasized their Indian roots and all participants' parents or grandparents had emigrated from India [23, 49, 50]. Thus, despite the fact that all participants had been born in Suriname, they felt closely related to India and particularly to Hinduism.

### 2.3. Recruitment Procedure

Participants were recruited using the “snowball” technique [51]. Community members for the individual interviews as well as the focus groups were recruited through two key informants and by the researcher, who visited group activities at the community center for ethnic minorities [52]. The first recruiter was a layman within the community and also the coordinator of the community center where, among other ethnic minorities, Hindus from all age groups organized cultural activities [52]. The second recruiter was a volunteer at a community center which coordinated a group of older Indian immigrants. Both recruiters participated in the focus groups, helping the researcher by clarifying participants.

Fifteen persons were approached for the individual interviews; seven rejected the invitation to participate due to busy schedules or lack of interest. In the focus groups none of the 19 participants rejected the invitation to participate. All members of the Hindustani immigrant community that were present during the recruitment periods and who were interested in the study received information about the study and gave their written informed consent. No incentives were given.

### 2.4. Data Collection and Study Procedure

Data were collected in the participants' natural environment since the goal of this study was to develop an understanding of the experiences of participants in their natural context [51]. Five participants preferred to be interviewed at the community center and three invited the researcher to their homes. Individual interviews were used to identify concepts that were relevant to the community members. During the focus group sessions, interactions were used to collect data about these concepts and confirm the results of the individual interviews [51].

The topic list for the focus group sessions emerged from the interview protocol and the individual interviews. The concepts of the TPB and HBM were applied by developing questions for each construct. In addition, questions regarding eating habits and PA were developed.

The interviews and focus group sessions were held in Dutch. All participants spoke Dutch fluently, although some participants (especially older participants) in the focus group discussions preferred to speak informally in “Hindi”.

The interviews and focus group discussions were audio taped and each lasted 60–90 minutes. Anonymity of all personal data was ensured by replacing participants' names by numbers before analyzing the data. Efforts were made to avoid paternalism by showing respect for the participants' culture. Finally, an attempt was made to reduce any psychosocial discomfort, which might have occurred during the interviews, by socializing with participants prior to the interviews and focus group sessions.

### 2.5. Data Analysis

First, all individual interviews and both focus group discussions were transcribed and double checked for accuracy of the transcriptions. Second, the data were entered NVivo and analyzed using a mixture of inductive and deductive methods. Categorization, based on the concepts of TPB, HBM, and the existing literature, was conducted a priori and described in a coding scheme. Each category was transformed into “nodes”. Quotes that reflected certain nodes were marked accordingly. Afterwards, data categorized under each node was reread and summarized. In this way important themes with regard to health beliefs about eating habits and PA were enlightened. Selected quotations were translated into English and checked by a professional language editor. Quotations from females were referred to as “F” and those from males by “M”, followed by their age, for example, a woman aged 52 is referred to as “F, 52”.

## 3. Results

In this section, the most important health beliefs that emerged from the data will be discussed. Questions based on the TPB and HBM revealed that attitude, perceived behavioral control, and intention to change were the most important concepts from the TPB, and cues to action was the most important concept from the HBM. First, the perceived behavioral control regarding health in general will be described; second, the specific health beliefs regarding eating habits and PA will be described.

### 3.1. Perceived Behavioral Control

At the beginning of the interviews and focus groups, respondents were asked to which extent they felt they could control their own health. Most respondents immediately emphasized responsibility for one's own circumstances, including health. In addition, respondents mentioned this belief was incorporated in the laws of karma, which is reflected by the following statement of a 55-year-old mother about her 27-year-old son:

*If he is ill… then I say “Well, God punishes immediately and fairly, so you probably did something wrong.” …that's karma, so, things will be weighed up, how many good things you did and how many bad things… (F, 55)*

On the other hand, when asked about the control over obesity-related consequences (e.g., CVD), genetics and chance-related factors were mentioned first:

*They say if it doesn't run in the family you won't get it… I have nobody in my family with cardiovascular diseases, so I don't have it yet. (F, 55)*

Thus determinants outside one's control (genes and chance) were emphasized when asking specifically about obesity-related consequences (such as CVD). However, in the course of the interviews, all respondents expressed that they were aware of the interplay between genes and lifestyle. Especially unhealthy diet and low PA levels were mentioned as related to unhealthy lifestyle:

*And sports, I think you can also use that to postpone diabetes and high blood pressure. As my husband has had a heart attack, and he went into rehabilitation, and they said the same: sports is just good, it's not that you won't get ill, but you can just postpone it by 10 to 15 years, and I find that too. (F, 48)*

### 3.2. Health Beliefs regarding Eating Habits

#### 3.2.1. Eating Habits: Attitude and Intention to Change

A diet with lots of sugar, carbohydrates, and fats was regarded as an unhealthy diet. Nevertheless, respondents expressed ambiguity about changing their eating habits; they seemed satisfied with their current behavior:

*When I go for my screening and I get a very different impression, then I might consider it [changing her diet], but… I think I'm on the right track… (F, 52)*

Respondent's ambiguity about changing traditional eating habits also was related to the importance of food in the life of Hindustani:

*Eating! That's number one [refers to the fact that on all social occasions, food plays a central role]! (F, 53)*

#### 3.2.2. Eating Habits: Perceived Behavioral Control

Several ways to reduce unhealthy eating habits were proposed: eating less “sugar-rich” basmati or pandan rice (replacing it by parboiled or unpolished rice, noodles, or potatoes), changing the way the rice was prepared (washing away the water after the rice was partly boiled), using sweeteners and eating less “prasad” (temple blessed food), changing the proportions of rice and vegetables (more vegetables, less rice), eating only one carbohydrate-rich product per meal instead of two (rice *or *potatoes, banana *or *bread), and using less oil or using olive oil instead of sunflower oil. 

Respondents also emphasized that traditional ways of preparing food were the only way Hindustanis had ever learned to cook and appreciate their food, and thus changing this behavior was difficult:

*I can go without [traditional, unhealthy, Hindu food], but only for a few days. (F, 52)*

Older respondents said that a cultural change was needed to change eating habits and were sure that for younger generation Hindustani immigrants, eating in a more healthy way was less difficult thanks to the larger variety of healthy foods currently available. To reduce the difficulty of eating healthier, older respondents expressed the need for culturally sensitive food advice:

*They don't have information about Hindu food… They're only talking about potatoes and stuff like that all the time. (F, 55)*

### 3.3. Physical Activity

#### 3.3.1. Physical Activity: Attitude and Intention to Change

Most respondents were convinced that PA offered benefits:

*…exercising I think can delay diabetes and high blood pressure. Because my husband also had a heart attack… his doctors also said exercising is simply good, not that it means you won't become ill, but you can just delay it by ten to fifteen years. (F, 48)*

In general, respondents acknowledged that their PA levels were low; almost all started laughing embarrassedly when they were asked about it. They reported walking to the supermarket or bus station, stretching, carrying out household activities, walking the stairs, and doing yoga for one hour a week. Only two out of the 27 respondents consciously worked on their health by exercising. However, these results might be influenced by age and gender, given that the majority of the women were in their fifties.

Surprisingly, most respondents also said they recently increased their PA level although they regarded PA as a necessity rather than a pleasant activity:

*I first ask what's it good for [exercise]?… I don't know anyone in my environment who really enjoys exercise… You do it because you have to, because the doctor prescribed it, especially when you have to pay [for exercising]. (F, 58)*

Group activities, such as dancing, swimming, and yoga, were regarded as the most enjoyable types of PA. 

#### 3.3.2. Physical Activity: Perceived Behavioral Control

Increasing PA was thought to be difficult due to a lack of personal supervision at sports centers, elderly Hindustani women felt ashamed to swim in front of men, the Dutch weather was too cold, and the costs of exercising were too high:

*…when you don't have the money, you can't pay. Look, as far as exercising goes, you can't go to the market every day, walking along the streets, especially the elderly… They would like to… Some say “It's too cold.”… some who want it aren't able to. (F, 53)*

### 3.4. Cues to Action

Respondents were mostly driven to improve their eating habits and increase their PA levels by the experience of obesity-related complaints such as CVD or diabetes themselves, within their family or through other direct personal contacts:

*My father-in-law also died at a very young age, 52. He had a severe form of diabetes… …well then you think “This could also happen to my husband and my children,” so that's why I became more aware. (F, 55)*

Other cues to action could be divided into advice from doctors (the family doctor or a rehabilitation specialist), brochures displayed at waiting rooms or the pharmacy, books, dieticians, colleagues, and sometimes husbands (only those husbands who were positive towards PA themselves). Furthermore, abnormal findings of blood sugar and cholesterol checkups motivated respondents to initiate healthier eating habits and increase PA levels. Other sources that were suggested to increase the motivation among Hindustanis were programs on the local radio presented by Hindustani role models or talks by pundits (Hindu priest).

## 4. Discussion

The nature of obesity varies across cultures, and preventing excess weight gain can therefore only be achieved by taking cultural background into account. Our study describes those cultural influences by assessing health beliefs regarding eating habits and PA to prevent obesity among the Surinamese Hindustani community in The Netherlands. These beliefs might have implications for the effectiveness of health education messages aimed at promoting healthy eating habits and increasing PA levels.

First of all, respondents argued that lifestyle influences health through karma. This is unique for Indian Hinduism and also is (partly) in contrast with previous studies [33–35, 38] in which cosmic (i.e., supernatural) illness causations were thought to affect some diseases but not CVD. Surprisingly, our study also indicated that CVD was attributed to factors within one's own control. This implies that cosmic illness causations do not necessarily hamper the interpretation of regular lifestyle advices; they are just another causal mechanism to explain health or illness. 

Second, we found that respondents in our study were aware of the preventive effect of a healthy diet and PA. This finding is in contrast to previous studies which found that Surinamese Hindustanis suggest different preventive measures to reduce obesity-related consequences (i.e., diabetes and CVD), such as not eating spicy foods [26]. This finding could be explained by the fact that most respondents in our study lived for decades in The Netherlands and therefore acculturation might have occurred. However, although the respondents in our study were aware of the health effect of a healthy diet and PA, our respondents suggested that more culturally sensitive information regarding typical Hindustani eating habits (“they are talking about potatoes all the time”) and women-only exercise facilities could improve health education messages. These results were also found by Farooqi et al. [27].

A third remarkable finding was that most respondents were convinced of their own ability to influence their health by improving their diet and increasing their PA levels but lacked the intention to do so. They expressed satisfaction with current behavior, which seemed ambiguous since most respondents experienced several important cues to action such as experiencing obesity-related complaints themselves (e.g., high blood pressure) or having known close peers that died from obesity-related diseases (predominantly CVD). This satisfaction with current eating habits and PA can be explained by the fact that immigrants attach a symbolic meaning to certain food traditions. They might (unconsciously) underestimate the relevance of changing some unhealthy food traditions in order to avoid incongruence between their ideal and current behavior. This “optimistic bias” [53] might thus be affected by the symbolic meaning those traditions have for immigrants [26, 31]. While food might be an important cultural element for Indians living in India, in a new country, where they might not be able to speak their native language and wear traditional clothing (regularly) anymore, traditional food and the preparation of that food become an important marker of identity [31]. This explanation is supported by the finding that most respondents emphasized they could not go without Hindu food for a few days, even though they knew it might sometimes harm their physical health. So, although health messages aimed at increasing awareness about personal susceptibility might be needed to overcome optimistic bias, due to the symbolic meaning of food for these migrants, this is not expected to result in any changes. The target group might argue that what might be considered bad for their physical health will be beneficial for their psychological health. Therefore, the combination of increasing risk awareness in combination with suggesting healthy alternatives for traditional eating habits is expected to be more suitable. 

Furthermore, in line with previous research [27–30], the influence of low levels of PA on excess weight gain and obesity-related consequences such as CVD and diabetes needs to be emphasized since the attitude towards PA and the intention to increase PA levels were found to be poor. Not only should lifestyle advice focus on the intensity of PA that is needed, but attention should also be given to the unfavorable attitude towards PA and perceived barriers for PA such as a lack of personal supervision at sports centers, shame of women to exercise in front of men, the weather, and costs. Farooqi et al. [27], who found similar results (the lack of women-only exercise facilities acted as a barrier for PA), suggests increasing the availability and affordability of women-only exercise facilities to overcome these perceived barriers [27]. Our study also indicated that providing information about access to moderately intense sports, guided fitness programs, or enjoyable PA classes such as dancing classes, yoga, and walking buddies for elderly Hindus might also stimulate this population to become physically active. Moreover, to remove another barrier to increase PA, lifestyle advice should recognize that most Hindustanis are not willing to pay high fees for such facilities. 

A potential limitation of this study regards the representativeness of the study sample; mostly middle-aged and older females and *Surinamese* Indian immigrants were included. However, the fact that mostly middle-aged women were included could also be considered a strength of this study since this particular immigrant population is affected the most by the obesity epidemic [3–5]. Furthermore, respondents described themselves as 100% Hindu and not as 100% Indian, which might indicate that respondents have (unconsciously) been affected by their stay in Suriname. However, 80.5% of all Indians consider themselves as Hindu [54] and therefore cultural implications related to Hinduism remain.

## 5. Conclusion

The current trend towards globalization and Westernization also affects the population of India and people of Indian descent living in other countries; they show increasingly sedentary lifestyles which leads to fast increasing numbers of obesity [1–6]. Since people of Indian origin are relatively more threatened by obesity-related consequences (i.e., CVD and diabetes), the need to develop and implement effective culturally tailored health education interventions for this group is urgent. Our study indicates that the Hindustani immigrants in our sample had consolidated their Hindu culture to such an extent that they are expected to benefit from culture-specific health education messages. To motivate this population to improve their eating habits and increase their PA levels, those advices could recognize the role of karma in explaining the onset of obesity-related consequences such as CVD, include more information about typical Hindu food products and preparation, and pay attention to the symbolic value of food for Hindustani immigrants. Finally, advices could suggest more enjoyable and culturally sensitive forms of PA for Hindustani women and could be delivered through pundits or radio shows.

## Figures and Tables

**Table 1:** Demographic factors of respondents.

	Individual interviews	Focus group 1	Focus group 2	Total
*N* participants	8	9	10	27
*N* males/females	2 M/6 F	1 M/8 F	10 F	3 M/24 F
Age range (mean) in years	29–58 (mean = 53)	47–82 (mean = 77)	42–63 (mean = 52)	29–83 (mean = 62)
% Employed	100%	0%	80%	60%
Connected through	Community house 1	Community house 2	Yoga class	—

**Table 2:** Health problems reported by respondents.

Health problems	High cholesterol levels	High blood pressure	Diabetes	Heart problems
*N* = 27	50%	10%	80%	70%
